# ‘All for the well-being of the infant’: nurses’ perceptions of preterm infants’ eye examinations: a phenomenographic study

**DOI:** 10.1186/s12887-024-05044-y

**Published:** 2024-09-13

**Authors:** Martina Carlsen Misic, Emma Olsson, Randi Dovland Andersen, Agneta Anderzén-Carlsson

**Affiliations:** 1 Department of Paediatrics, Faculty of Medicine and Health, Örebro University, Örebro, Sweden; 2 Faculty of Medicine and Health, School of Health Sciences, Örebro University, Örebro, Sweden; 3 Research Centre for Habilitation and Rehabilitation Models & Services (CHARM), Faculty of Medicine, University of Oslo, Oslo, Norway; 4 Department of Research, Telemark Hospital Trust, Skien, Norway; 5 University Health Care Research Centre, Faculty of Medicine and Health, Örebro University, Örebro, Sweden; 6 Faculty of Health and Social Sciences, Inland Norway University of Applied Sciences, Elverum, Norway

**Keywords:** Neonatal, NICU, Nursing, Premature, Preterm, ROP screening

## Abstract

**Background:**

Preterm infants are at risk of complications due to their prematurity and Retinopathy of Prematurity (ROP) is one of them. To discover and treat ROP the preterm infants regularly undergo eye examinations. Nurses are responsible for the infants’ care during this painful and stressful procedure.

**Aim:**

The aim of this study was to explore nurses’ perceptions of preterm infants’ eye examinations.

**Methods:**

Data were collected through semi-structured interviews with 10 nurses experienced in participating in preterm infants’ eye examinations. Data were analysed using a phenomenographic approach.

**Results:**

The results showed several perceptions of the eye examinations, and the analysis resulted in four descriptive categories: *Infants are affected by the eye examination*; *Nurses have comprehensive overall responsibility for the infants*; *Parents are important to their infants*,* but they need support to fulfil their parental role*, and *Collaboration is important for the examination’s favourable outcome.* The category *Nurses have comprehensive overall responsibility for the infants* was regarded as the most comprehensive, covering all the other categories.

**Conclusions:**

Nurses felt a great responsibility during a painful and stressful procedure for preterm infants. Infants’ well-being could be better protected by interprofessional collaboration, improved nursing care and involved parents.

## Background

Preterm infants are born before they are ready to live outside the womb. The earlier the infant is born, the higher the risk of various complications [1]. One of those is retinopathy of prematurity (ROP), a disease that affects the immature retina and its vessels. The eye should normally develop in the oxygen-deficient environment in the uterus, but with preterm birth the eye is exposed to increased and altered levels of oxygen, which can cause a pathological development of the retinal vessels [2].

According to Swedish guidelines, the fundus of the eye is examined in all infants born before 30 weeks gestational age to discover and treat ROP [3]. The examinations are performed regularly until the infants reach full term or the retina is fully developed. The frequency of examinations depends on the stage of ROP and the development of the eye [3]. In Sweden, these eye examinations are performed using either indirect ophthalmoscopy or retinal imaging. During indirect ophthalmoscopy, the eyes are held open manually or with an eyelid speculum, and a bright light is pointed into the dilated pupil while the ophthalmologist examines the eye with an ophthalmoscope. During retinal imaging, the eye is held open by eyelid speculums and the fundus are photographed for later diagnostics [4]. The eye examination can take place in the infant’s care room, an examination room, or the ophthalmology unit depending on the required level of care. Usually, health care professionals from both the neonatal unit and the eye clinic and the parents are present at the examination to support the infant.

To facilitate the retinal examination, infants receive mydriatic eye drops to dilate the pupils before the eye examination. Side effects can be cardiovascular, gastrointestinal, and respiratory [5]. When eyelid speculums are used, the infant usually receives pain-relieving eye drops before the examination [4].

The eye examination consists of stressful and painful elements for the infant, including being held in position and subjected to bright light and sometimes physical manipulation of the eye [6]. Despite extensive research into pharmacological, physical, psychological, and multimodal methods to alleviate infants’ pain during eye examinations, no effective treatment has yet been identified [7–11].

Nurses at the neonatal unit are responsible for the preterm infants and supervise their care. Nurses have been shown to experience stress when infants receive inadequate pain relief during procedures and when they are responsible for an infant’s distress. Neonatal nurses have also expressed emotional distress when trying to protect an infant from pain and discomfort [12]. Individual nurses’ perceptions of suffering may also affect how nurses experience infants´ suffering during a procedure [13]. However, perceptions and attitudes towards painful procedures also depend on nurses’ knowledge and awareness of infant pain and preterm infants´ pain signals [14]. In particular, the smallest infants’ pain can be overlooked and undertreated, since their pain expressions can be very subtle [15].

In summary, despite research showing that preterm infants experience pain and distress during eye examinations, no adequate pain management is yet available. Although nurses are responsible for infants’ care and pain assessment, little is known about their experiences during the eye examinations. A better understanding of how nurses perceive infants’ eye examinations could help identify areas for improvement in care during eye examinations and potentially lead to more effective pain and stress management.

### Aim

The aim of this study was to explore nurses’ perceptions of preterm infants’ eye examinations.

## Methods

### Design

In this phenomenographic interview study, the phenomenon under study was preterm infants’ eye examinations. A phenomenographic approach aims to describe the various ways people experience and comprehend a phenomenon in the world around them [16]. This is also referred to as a second-order perspective [17]. While a first-order perspective would provide a description of the various aspects of infant eye examinations (how something “really is”), a second-order perspective provides a description of how the different aspects of infant eye examinations were perceived by the participating nurses. The reporting of the Study followed The Consolidated Criteria for Reporting Qualitative Research (COREQ) [18].

### Study setting and recruitment

Nurses working at Swedish neonatal units with > 12 months neonatal work experience and who had participated in at least two eye examinations of preterm infants were invited to participate in the study. Information about the study was distributed to various organizations for paediatric nurses, Facebook groups for Swedish neonatal nurses, and personal contacts of the authors. A snowball selection strategy [19] was also used where participating nurses could suggest colleagues who might be qualified and interested in participating.

### Data collection

To capture qualitative descriptions of the participants’ experiences [20], semi-structured interviews were conducted from February 2021 to May 2022. An interview guide was developed from a version used in a previous study addressing nurses’ experiences of caring for non-verbal children in pain [21]. In the current study, the questions were adapted specifically to preterm infants and eye examinations. In preparation for the interview, the nurses were asked to recall two eye examinations they had participated in, preferably one they perceived as positive and one they considered unfavourable. A pilot interview was performed to test the interview guide. No changes were made to the guide and the pilot interview was included in the analysis.

All interviews but one were performed online due to COVID-19 restrictions. The first author performed eight interviews, and the last author performed two interviews with nurses who had ongoing professional relationships with the first author. Both interviewers are paediatric nurses with neonatal care experience. The last author is an experienced qualitative researcher, and the first author is a doctoral student. The interviews, which took from 17 to 60 min (median = 34 min) were recorded on digital voice recorders and transcribed by the first author.

### Characteristics of participants

Ten neonatal nurses from seven different hospitals volunteered to participate and were included in the study. Their ages ranged from 27 to 65 (mean 42 years) and their working experience in neonatal intensive care ranged from 5 to 38 years (mean 17 years). Nine nurses worked at a university hospital and six of the seven university hospitals in Sweden were represented. Only female nurses chose to participate. Nine had a degree in paediatric nursing and one was undergoing specialist education at the time of the data collection.

### Data analysis

The data analysis followed the steps described by Sjöström and Dahlgren [17], which are further described in Table 1. Although the steps are described linearly, the analysis was iterative and moved back and forth among the steps.

**Table 1:** The seven analytical steps described by Sjöström and Dahlgren (2002) as performed in the current study

Familiarization	The first author listened to the interviews and corrected the transcripts. All authors read the transcribed interviews to familiarize themselves with the data.
*Compilation of responses*	The most significant parts of the informants’ responses were identified and extracted from four of the transcripts. Each author extracted significant parts individually, and these were compared within the group to confirm that similar parts were identified. The first author then extracted and compiled significant responses from the remaining transcripts.
*Condensation*	The individual answers were shortened to identify their most central parts: the first author condensed the excerpts, and the co-authors read through the condensed material, compared it with the original statements, and discussed their understandings to ensure that the substantial meanings were retained.
*Preliminary grouping*	The first author sorted the condensed meanings into preliminary categories, which all authors discussed on several occasions.
*Comparison of categories*	The preliminary categories were compared to find associations and establish borders between them. The authors discussed and revised these associations and borders in a process to agree on subcategories and descriptive categories.
*Naming of the categories*	After thoroughly discussing their collective understanding of the categories, all authors named the final descriptive and subcategories to highlight their inherent meanings.
*Contrastive comparison*	The unique character of each descriptive category and the resemblances between them were described.

## Results

**Fig. 1:**
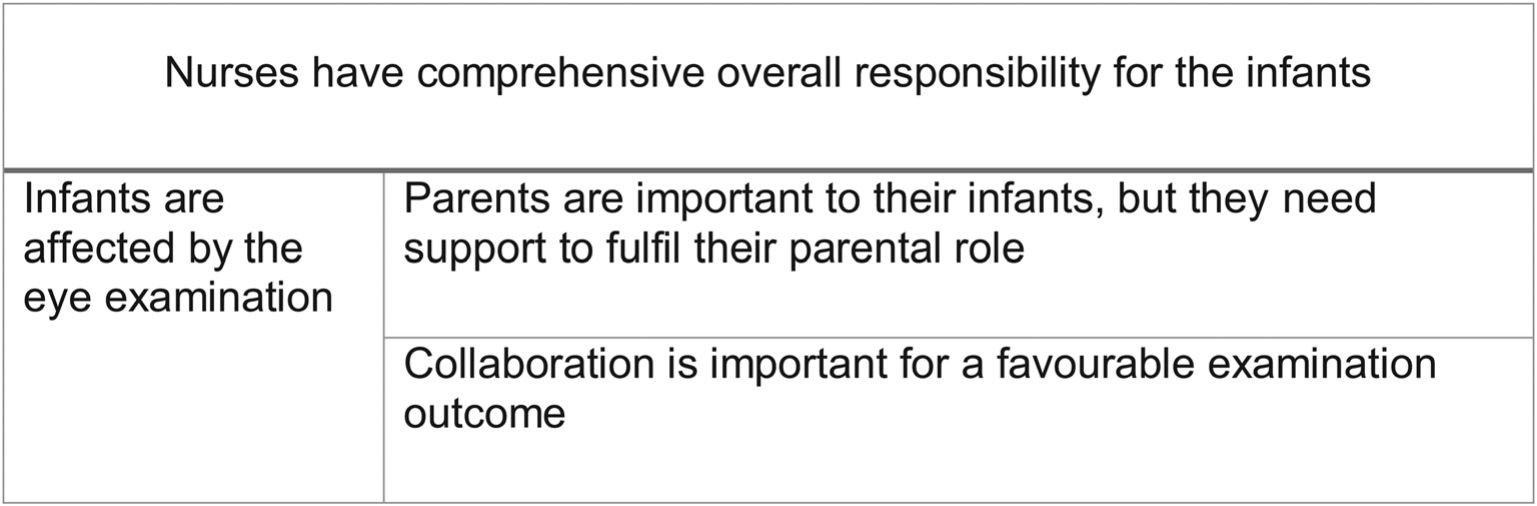
Hierarchy of and resemblance between the descriptive categories

The nurses’ perceptions of the preterm infants’ eye examinations resulted in four descriptive categories: [1] *Infants are affected by the eye examination*; [2] *Nurses have comprehensive overall responsibility for the infants*; [3] *Parents are important to their infants*,* but they need support to fulfil their parental role;* and [4] *Collaboration is important for a favourable examination outcome.* The resemblances between the categories are described in Fig. 1 and in Table 2 the categories and respective sub-categories are outlined.

### Contrastive comparison of the categories

The descriptive categories showed a variety of nurses’ perspectives of the phenomenon under study, all of which were related to the nurses’ focus on the infant’s best interest. Nurses considered the infant the central figure in the eye examination, and they perceived their primary professional role as advocating for the infant and safeguarding their well-being. The eye examinations were perceived to cause infants distress and discomfort, and nursing care was provided to support the infants’ well-being. Despite the necessity of nursing care, parents were recognized as the most important people to the infant, but the parents required support and guidance from the nurses and the ophthalmologists to take an active role. To achieve the best outcome for the infant, interprofessional and parental collaboration was necessary, which in turn required a shared understanding of the infant’s well-being. The nurses perceived themselves as indispensable in facilitating this collaboration. Inherent in all the perspectives of the phenomenon is the nurses’ implicit notion that nurses have comprehensive overall responsibility for the infants, which is the most complex descriptive category. This includes to safeguard the wellbeing for the infant and parents in the here and now, while simultaneously facilitate for the ophthalmologist to be able to perform the examination accurately, as this is of crucial importance for the infant’s future health. Furthermore, there is a relationship between the descriptive category “Infants are affected by the eye examination” and the descriptive categories “Parents are important to their infants, but they need support to fulfil their parental role” and “Collaboration is important for a favourable examination outcome” respectively. In addition to nursing care, both parental support and collaboration are important to ensure the wellbeing of the infant.

**Table 2:** Descriptive categories and subcategories

Infants are affected by the eye examination	Nurses have comprehensive overall responsibility for the infants	Parents are important to their infants, but they need support to fulfil their parental role	Collaboration is important for a favourable examination outcome
The examination causes the infant discomfort	Nurses want to be in control to safeguard the infant’s well-being	The examination is stressful for parents	Communication and joint understanding are crucial
Infants react differently during the examination	Nurses provide infants with nursing care and administer pharmacological treatment	Parents need information and support to assist their infant during the examination	The attitude of the ophthalmologist affects the atmosphere of the examination
	Nurses advocate for the infant	Parents support their infant in different ways	
	Nurses become emotionally involved		

### Infants are affected by the eye examination

#### The examination causes the infant discomfort

Based on how the examination looks and how the infants react, nurses perceived the eye examinations as unpleasant for the infants. It was also described as stressful and challenging. Even if the infant managed the examination well, it was still perceived as a difficult situation.

I do not know if I have ever seen an ROP examination that has been top notch since they are tough. (Nurse 3)

Some nurses explicitly mentioned the examination as painful for the infants, particularly when the eye was directly manipulated or when a speculum was used to hold the eye lids open. The use of the speculum was described as “horrible” and “uncomfortable” for the infants.

The perceptions of different examination methods varied, and although the ‘horrible’ eyelid speculum was used during the RetCam examinations, some nurses recognized this method as more convenient for the infant, as it led to quicker and safer examinations.

The challenging part [of the eye examination] takes approximately thirty seconds in an eye with severe ROP changes. So, thirty seconds or ten minutes, you can understand that the [shorter] time perspective is the winner [comparing the time to use RetCam with conducting a manual examination]. (Nurse 4)

### Infants react differently during the examination

The infants displayed different reactions both during different stages of the eye examinations and in comparison to other infants. However, common reactions during the examinations were changes in physiological parameters such as a reduction in oxygen saturation and heart rate. Infants could also require more oxygen throughout the examinations.

His oxygen saturation fluctuates a lot due to his very sick lungs, but during the examination it becomes even worse. (Nurse 3)

Nurses also observed different behavioural expressions of discomfort, such as crying, tensing arms or legs, or becoming ‘floppy’. Nurses also formed an impression of the infant as stressed.

I knew her well and noticed that she was twisting and got a bit distressed. She moved her arms. Despite holding her, one could feel that she was restless. (Nurse 10)

Different factors affected how the infant responded to the eye examinations; some nurses mentioned the infants’ levels of sickness and maturity, and they noted that the same infant could react differently to examinations at different times.

Well, my experience is that infants react during eye examination on different levels, but there is always some sort of reaction. (Nurse 1)

After the eye examination, the infants were often perceived as tired and in need of recovery time, especially after examinations that nurses regarded as unfavourable.

And you can also see that the infants are exhausted afterwards, they are unstable. (Nurse 5)

It was not only the examination that was perceived to affect the infant’s well-being. The eye drops given before the examination could also have negative side effects, such as increased heart rate, digestive problems, and sensitivity to light.

Despite the general perception that the infants usually reacted negatively to the examination, there were also reports of infants who did not seem to react. Some infants slept through the examination or were perceived as calm and physically stable.

She was calm and stable regarding oxygen saturation, heart rate, and blood pressure, and she was not at all affected during the examination. (Nurse 9)

### Nurses have comprehensive overall responsibility for the infants

#### Nurses want to be in control to safeguard the infant’s well-being

The nurses felt responsible for the infants’ well-being during the examination since they regarded themselves as knowing the infant best. They wanted to be in control of the infant’s status, both by controlling equipment such as respiratory support and monitoring the infant’s physiological parameters and expressions.

I trust myself, and I also feel that I am [the one responsible]. If, for example, the baby is on CPAP or a ventilator, then I want to be in control to ensure that everything is still in place and the mask has not slid out of position. I want to be there [in a central position], so I really can see and not have the [ophthalmologist’s] hands in the way. (Nurse 8)

### Nurses provide the infant with nursing care and administer pharmacological treatment

Nursing care was perceived as crucial to the infant’s ability to cope with the examination. The nurses believed that their nursing care could make a significant difference for the infant during the examination and that such care helped the infants get through the examination. The goal of their nursing care was to leave the infant with enough strength to recover after the examination.

Because of our different understanding of the infants and their needs for support and holding [compared with professionals from the eye clinic], we can help and comfort them during the very tough situation of the eye examination. This is true whether the ophthalmologist uses lights only or uses RetCam: one can make a great difference solely with nursing care. (Nurse 7)

Preparations before the eye examination were recognized as an essential aspect of nursing care and an important prerequisite for the infant’s well-being during the examination. These could include swaddling, changing the diaper, and feeding. During the examination, holding the infant was described as crucial to assist the infant and to make them feel safe and supported through the examination. Despite this positive aspect of holding, it could sometimes be perceived as restraining the infant, especially when it was important for the infant to keep still for the examination to be safe.

Another aspect of nursing care considered important for the well-being of the infant was their positioning. Most infants disliked being placed on their backs during the examination. By positioning infants on their side, even more seriously ill infants could get through the eye examination while remaining relatively stable. The nurses also said it was important to take breaks when the infant showed signs of exhaustion, decreased oxygen saturation, or low pulse during the examination. Infants were given extra support after the examination, such as by being held skin-to-skin by a parent or by having fewer other procedures the same day.

The infant’s perceived experience of the examination as hard and stressful could lead to a change of routines. By changing the place of the examination to the arms of the parents, for example, the nurses experienced an immense improvement for the infants. This led to nurses having more positive feelings towards the examination and perceiving that the infants handled the examination better and recovered sooner. *‘We are so positive now; we think that it goes so much better and that feels great’* (Nurse 5). Nevertheless, it was also perceived as important that the examination should be safe for the infant as well as ergonomic for the ophthalmologist.

Adaptations of the environment such as dimmed lights and a reduction of noise in the room where the examination took place were other nursing care actions to support the infants. The nurses always tried to keep a calm and quiet environment, but this was perceived as especially important during eye examinations. By reducing the lights, nurses felt that the activity in the room automatically slowed down. As the infants were perceived as sensitive to light due to the mydriatic eyedrops, the room was kept dark after the examination as well. Although the infants were given optimal conditions, however, the nurses sometimes perceived that it was not enough to support them.

We swaddle, we give [oral] glucose, we help by holding the head, the setting is quiet and calm, but the infant is still not settled, maybe unstable in oxygen saturation, screams, and is sad and sweaty. (Nurse 6)

Besides nursing care, the nurses also supported the infants by administering pharmacological treatments. The infants sometimes received oral glucose before and during the eye examination and in combination with a pacifier. Other pharmacological support, such as sedatives or analgesics were not prescribed routinely, but were offered to infants when needed. Some nurses debated the necessity of providing more pharmacological support during the eye examination.

### Nurses advocate for the infant

The nurses considered themselves as the voice of the infants. As such, it was their obligation to advocate for the infant during the eye examinations. When an infant was not doing well, they spoke up and stopped the ophthalmologist from proceeding with the examination.

I noticed the condition of the infant and spoke up. I acted as the child’s advocate; it feels like my most important role in such a situation. (Nurse 9)

There was a strong belief among the nurses that the parents of the infant should be present to support their infant during the eye examinations. They believed that the role of the parents was irreplaceable, and they therefore worked to enable the parents to be there for their infant even when it was difficult for the parents to cope with the situation.

### Nurses become emotionally involved

The eye examinations affected the nurses emotionally. In general, they were perceived as a troublesome procedure for the infants. When the examinations were perceived to go smoothly and favourably for the infant, the nurses felt content and satisfied.

It is for the infant. I want the infant to be content, and when I notice that the infant is doing well, then I feel well, too. (Nurse 8)

The nurses wanted to do what was best for the infants, but although they did their very best, they felt it was not enough if the examination turned out to affect the infant badly. Nurses also felt frustrated when they felt the ophthalmologist did not listen to them and went ahead with the examination in a way that was not in line with the condition of the infant.

When the examination was perceived as unfavourable for the infant, nurses could have feelings of failure. They empathized strongly with the infant in discomfort, and it was difficult for them to see the infants crying and suffering. Nurses usually felt sad after examinations they perceived as having caused the infant to suffer, and they tried to cope with this by helping the infant and parents with extra support afterwards.

It tears you up; you almost feel it yourself when they start having decreases in their oxygen levels and do not feel well afterwards. I feel it in my whole body, because when you work with these [vulnerable infants] you want to do what is best for each infant. (Nurse 7)

Because the examination could cause pain and discomfort to the infant, the nurses sometimes questioned the necessity of the eye examinations, but mostly they agreed that it was an important and necessary procedure for the sake of the infants’ long-term well-being.

### Parents are important to their infants, but they need support to fulfil their parental role

#### The examination is stressful for parents

The examinations were perceived as difficult for the parents to endure and sometimes the parents could not manage to be present. When the examination made the infant suffer, parents were perceived as feeling sad and miserable, which made it hard for them to support their infant.

I have experienced, during an unfavourable examination that they [the parents] can’t stand staying at bedside and instead they say ‘I´ll go and sit over there for a while’, the staff stands closest to the infant instead. (Nurse 5)

### Parents need information and support to assist their infant during the examination

Nurses felt it was important that parents were informed about the examination to be able to participate and support their infant. They also considered it essential to prepare parents for what to expect during the examination. The nurses felt they had more knowledge and control than the parents and so they could predict how the infant might react during the examination. They perceived it as important to share this knowledge with the parents to support them in being active participants in the care of their infants. Parents were informed about how the examination was performed, how they could provide support, and how their infant might react during the examination. The parents also received information about the findings in the eyes directly from the ophthalmologist before, during, and after the examination, which the nurses felt was beneficial.

The nurses’ perceived that it was important to encourage the parents by telling them that they had an important role in supporting the infant. For the well-being of the parents, the nurses felt it was better for the parents to see the examination with their own eyes than to have it described to them afterwards.

To recite the course of the examination to them feels totally worthless. They need to see what is done to their infants. (Nurse 1)

### Parents support their infant in different ways

When the parents were informed and had previously participated in eye examinations, they could take on a more active parental role in supporting their infant. As parents gained experience in participating in the care of their infant, nurses perceived that providing support during the examination came more naturally to them.

The nurses described how the parents comforted their infants by holding them and talking or singing calmly. Occasionally parents could also administer the oral glucose and offer the pacifier to the infant. Because nurses felt that the parents should be the infants’ main support during the examination, they were willing to change their nursing role to support the infant through supporting the parents.

When the examination was performed in the arms of the parents, the nurses perceived it as positive for both the parents and the infants. The parents then became more included and took a more active role supporting the infant. The nurses felt that the infants became much calmer, which shortened the examination time.

Even if it is a challenge and exhausting for the infant – challenging because an eye examination is still a tough procedure – they recover more rapidly when comforted and supported by their parents. (Nurse 4)

Likewise, the nurses experienced it as more favourable for both infants and parents when the examinations took place in the family care room. The infant did not have to be moved into another room, and the parents were perceived to feel safer in the environment they had created with their infant at the unit.

Previous experiences during the neonatal care could affect the parents in ways that the nurses perceived as negative for the infant. This could cause a clash in perspectives between what nurses and parents thought the infant needed.

#### Collaboration is important for a favourable examination outcome

##### Communication and a joint understanding are crucial

Collaboration was described as positive interaction between nurses, parents, and ophthalmologists to perform the examination in the best possible way for the infant. Communication was crucial to the interaction between the nurses and the ophthalmologists, and by sharing information they could create a joint understanding of the infant’s situation. Since the ophthalmologists did not have primary knowledge about preterm infants, the nurses considered themselves responsible both for the infant’s well-being during the examination and for leading the team. The nurses expressed a need for ophthalmologists to learn how best to care for preterm infants and improve their well-being during the examination.

The collaboration around the infant during the examinations also included other colleagues. When the infants were more fragile and vulnerable, more staff were needed to support them. In such cases an explicit distribution of responsibilities was agreed upon; for example, one staff member might be responsible for keeping the breathing tube in place, another for monitoring the infant’s physiological measures, a third for supporting the parents and the infant, and a fourth for assisting the ophthalmologist by holding the infant’s head.

The nurses believed it was favourable when the ophthalmologists brought their own assistant from the eye clinic to help them. Nevertheless, sometimes the nurses regarded themselves as the ophthalmologist’s assistant by being responsible for creating favourable conditions for the examination.

The nurses’ experiences of collaborating with ophthalmologists were mostly favourable.

However, there were times when the interaction was experienced as unfavourable.

The triangle of us [the nurse, the ophthalmologist, and the parent] does not really come together to help the infant. So, the collaboration between the three of us did not work optimally. (Nurse 2)

#### The attitude of the ophthalmologist affects the mood of the examination

The nurses mostly reported the ophthalmologists as acting professionally and responding to the infant’s well-being. The examining ophthalmologists were often known to the nurses from previous examinations, which generated a feeling of security.

It was generally perceived as negative when the different professionals’ perspectives collided, for example, when nurses and ophthalmologists did not have a shared understanding of the examination situation. The examination could also be perceived as less favourable for the infant when nurses felt the ophthalmologist did not listen to them and prioritized finishing the examination over the infant’s well-being.

He was not very cautious and did not listen [to the nurse]. He just wanted to turn up and do what he was supposed to do and then leave. It felt like he didn’t give a shit about whether it was okay for the infant or not. (Nurse 10)

When the nurses wanted to further develop their caring routines during the eye-examinations, the ophthalmologists were not always positive about the change and questioned whether the examination could still be performed correctly and whether the infant’s well-being was more important than the outcome of the examination. However, some ophthalmologists were reported to support the development of new routines for the well-being of the infant. In addition, the nurses talked about ophthalmologists who themselves had initiated to perform the eye examination with the infant placed skin-to-skin with one of their parents.

The nurses expressed a desire for the ophthalmologist to perform the examination quickly and with infant’s best of interest in focus. However, sometimes they observed that ophthalmologists were stressed by a large workload and having many infants to examine within a limited time. The stress caused by these circumstances was perceived as negative for the outcome of the examination, as it threatened the infant’s well-being, especially when the ophthalmologist did not want to take breaks that the infant needed. However, some nurses considered that earlier negative experiences could affect the ophthalmologists’ understanding about why an examination might need to be stopped.

## Discussion

Nurses found ROP eye examinations to be uncomfortable and stressful for the infant. Their overall goal during the eye examination was to support the well-being of the infant and they considered nursing care, which was their major responsibility, very important. They also emphasized the importance of the parents as an irreplaceable support for the infants during the procedure. A favourable examination for the infant required collaboration among the professionals and the parents. The ophthalmologist’s attitude was also reported as essential to the success of the procedure.

### Infants are affected by the eye examination

The nurses described some elements of the examination, such as the use of the eyelid speculum and manipulation of the eye as painful, but not the examination itself. Instead, they used words such as “discomfort” or “stress” when describing their experiences of infants undergoing eye examinations. This is somewhat different from previous research, which on a more general level has claimed that eye examinations could be painful for the infant [6]. Perhaps the thought of participating in a routine procedure that causes pain is challenging for nurses, making them use less loaded expressions when describing their perception of the infants’ experience as a coping strategy. However, such coping mechanisms could lead to bias in their assessments of infants’ pain [22]. Previous research indicates underassessment of infants’ pain may be due more to factors such as the infants’ inability to express pain or previous knowledge of the infants from other caring situations [23] than to nurses’ biases. Since infants are not able to self-report their pain and distress, other indicators are needed. Korhonen et al. [13], suggested the use of physiological parameters such as alterations in oxygen saturation and heart rate along with behaviours such as crying and moving. The nurses in this study described how such indicators of pain helped them to interpret when the child was in pain, adding to our knowledge on pain associated with eye examinations in preterm infants.

### Nurses have comprehensive overall responsibility for the infants

As in other previous studies of the nursing role during ROP examinations of preterm infants, these nurses felt an overall responsibility for the outcome of the eye examinations. In fact, it was found to be the most comprehensive way of thinking about eye examinations. The nursing role has been identified as preventing ROP through nursing care and taking responsibility for the infant before, during, and after the eye examinations, and supporting and counselling the families [24]. However, the particular role and overall responsibility of the neonatal nurse is complex and remains not only during eye examinations. Nurses in neonatal units need to have special competencies that are crucial for providing safe and high-quality care [25].

The nurses in this study became emotionally involved and mentioned feelings of failure when they perceived distress in the infants as they considered preventing such distress a part of their overall responsibility. This is comparable to earlier research in which nurses expressed feelings of stress and personal pain when they felt the infants in their care were not doing well [12]. These findings indicate a need for further investigation into the stress of neonatal nurses to identify what actions might be taken to prevent and alleviate these feelings.

### Parents are important to their infants, but they need support to fulfil their parental role

The parents were perceived as invaluable partners during eye examinations. This could be because parents are now usually recognized in neonatal care units as primary caregivers to their infants. Several pillars of family-centred care in this setting, such as the principles of information-sharing, support, collaboration, and empowerment, are mentioned in the findings of this study. In family-centred care, parents are supported in caring for their infants, usually with positive outcomes for both the infants and the parents [26]. When the parents are present in the neonatal unit and regarded as members of the team, they become even more involved in the care of their infant and take on a more active role [27], which was in line with the current findings. Involving parents has also been found to improve the infants’ pain management [28], and parents want and need to participate in their infants’ pain management [29]. The nurses in the current study strongly supported the positive effect of parental involvement in their infant’s care and pain management, but they also reported that some parents could not manage being at the eye examinations. Therefore, supporting parents to stay at their infant’s bedside was another aspect of the nurses’ responsibility to provide the best circumstances for infants undergoing eye examinations, as outlined in this descriptive category.

## Collaboration is important for a favourable examination outcome

The setting of the eye examination includes interprofessional collaboration between the nurses and ophthalmologists, but the nurses highlighted that the collaboration needed to be based on a mutual understanding and respect for each other’s competences. This is in line with theoretical framework on interprofessional collaboration, where collective action has been called for to address complex tasks and for team members to respect and trust each other [30].

Ophthalmologists in a previous qualitative study expressed discomfort with examining preterm infants and reported that stress and the set-up of the examination could make it burdensome. Nevertheless, they said they were committed to the task and needed the nurses’ help with preparations for the examination [31]. When the nurses in the current study discussed their collaboration with the ophthalmologists, they demonstrated an understanding of these aspects. Neonatal care includes several types of interprofessional collaboration, both in the daily work at the unit and in other units; therefore, these nurses have much experience of collaboration. In the future, however, the particular topic of collaboration during eye examinations could be explored more in detail to facilitate collaboration focused on the best interests of the infant.

### Implications for education

The various ways of understanding preterm infants’ eye examinations, can provide a base for nurses’ learning and education. Nurses need knowledge and skills within the areas identified in this study. They need knowledge and education about pain and how preterm infants express pain, the nursing care and pharmacological treatment during eye examinations and how to best support parents in these situations. Furthermore, they need knowledge about the ophthalmologist’s procedures and experiences from performing eye examinations, as well as knowledge and skills in how to best facilitate collaboration. All this knowledge is needed for the wellbeing of the infant.

### Strengths and limitations of the work

A major strength of this study is its focus on the nurses’ perspectives on eye examinations in preterm infants, which adds to our knowledge of various professionals’ perspectives. The nurses in this study worked in different hospitals and had different experiences, which is another strength as it allowed us to capture different perspectives on, and perceptions of, the phenomenon under study. The sample size was rather small, but nevertheless, we obtained a variation of perceptions, adding to the opportunity for a contrastive comparison of the descriptive categories and to find a resemblance between them. In phenomenographic research a range of 10–30 participants are typically considered large enough to capture sufficient variation [32]. In the future, it would be valuable to add to current knowledge by researching the perspectives of parents and other staff at the neonatal unit. As the phenomenographic approach strives to investigate the phenomenon within a homogeneous group [33], we did not consider it appropriate to include other perspectives in this study.

The nurses were asked to prepare themselves before the interview by thinking of two cases they had experienced, preferably one positive and one negative. This design created variation within the phenomenon, which is desirable in a phenomenographic design and could be seen as a strength [16]. However, not all nurses imagined individual cases, but reported more generally on what they perceived as more or less favourable eye examinations, which could be considered a limitation.

The first author, who performed most of the reviews had limited experience of interviewing, which could be regarded as a limitation; however, the interview guide was thoroughly discussed, and the interviews were continually reviewed and followed up on by the other researchers to control the quality of the interviews and to supervise the interviewer, which increases the credibility of the study.

Despite some researchers stating the importance to adhere to the advances in methodology [34], we decided to follow the analysis steps by Sjöström and Dahlgren [17], which for example differ from more recent approaches, as they do not use the concept of ‘outcome space’. Instead, the last step in the approach described by Sjöström and Dahlgren [17] is to describe the unique character of each descriptive category and the resemblances between categories. Nevertheless, we interpret that as being similar to what in later research is referred to as an outcome space. Sin [33] for example, states that an outcome space is accommodating both the unique essence of each category and the relationships between them [33]. In this article the relationship and hierarchy between the categories was presented in Table 2; Fig. 1.

#### Recommendations for further research

Neonatal nurses participate in advanced care of preterm infants and assist in various painful and stressful procedures. Further research is therefore needed to explore how nurses experience these situations and to further develop the nursing care and support these nurses. The perspectives of other members in the team around the infant are also of interest for future research; since the perspectives of ophthalmologists have already been studied, there is a need for more knowledge about the parents’ experiences. Furthermore, as previous research has shown that eye examinations generate pain and stress in infants, additional research is needed to improve nursing care of the infants during these examinations. Future research might also include an observational study using video technology to capture infants’ expressions of pain during different phases of the examination, the work of the nurses, and the collaboration among all parties present.

#### Implications for policy and practice

The nurses’ reported perceptions showed that infants’ pain might vary during eye examinations, that the parents’ presence was important, that the nurses felt a huge responsibility, and that a well-functioning collaboration is needed for a favourable outcome for the infant. This knowledge is of great value for nurses, but also for ophthalmologists and other health care professionals participating in eye examinations of preterm infants. These results may also be applicable to other painful procedures performed in neonatal care. Furthermore, the findings bring useful insights for managers. If they are to develop the care of preterm infants during eye examinations, they need to consider various aspects of care and support for all involved parties.

## Conclusions

The nurses in this study perceived themselves as having overall responsibility for preserving the well-being of the infant and for the eye examination to be successful. By providing nursing care, supporting parental participation and by interprofessional collaboration they believed that the well-being of the infant could be safeguarded.

## Data Availability

The datasets used and/or analysed during the current study are available from the corresponding author on reasonable request.
